# 17β-Estradiol Modulates SIRT1 and Halts Oxidative Stress-Mediated Cognitive Impairment in a Male Aging Mouse Model

**DOI:** 10.3390/cells8080928

**Published:** 2019-08-19

**Authors:** Mehtab Khan, Rahat Ullah, Shafiq Ur Rehman, Shahid Ali Shah, Kamran Saeed, Tahir Muhammad, Hyun Young Park, Myeung Hoon Jo, Kyonghwan Choe, Bart P.F. Rutten, Myeong Ok Kim

**Affiliations:** 1 Division of Life sciences and Applied Life Science (BK 21plus), College of Natural Science, Gyeongsang National University, Jinju 52828, Korea; 2 Department of Psychiatry and Neuropsychology, School for Mental Health and Neuroscience (MHeNS), Medical Center (MUMC+), Faculty of Health, Medicine and Life Sciences, Maastricht University, European Graduate School of Neuroscience (EURON), 6229ER Maastricht, The Netherlands

**Keywords:** d-galactose, 17β-Estradiol, SIRT1, ROS, neuroinflammation neurodegeneration

## Abstract

Oxidative stress has been considered the main mediator in neurodegenerative disease and in normal aging processes. Several studies have reported that the accumulation of reactive oxygen species (ROS), elevated oxidative stress, and neuroinflammation result in cellular malfunction. These conditions lead to neuronal cell death in aging-related neurodegenerative disorders such as Alzheimer’s disease (AD) and Parkinson’s disease. Chronic administration of d-galactose (d-gal) for a period of 10 weeks causes ROS generation and neuroinflammation, ultimately leading to cognitive impairment. In this study, we evaluated the estrogen receptor α (ERα)/silent mating type information regulation 2 homolog 1 (SIRT1)-dependent antioxidant efficacy of 17β-estradiol against d-gal-induced oxidative damage-mediated cognitive dysfunction in a male mouse model. The results indicate that 17β-estradiol, by stimulating ERα/SIRT1, halts d-gal-induced oxidative stress–mediated JNK/NF-ҡB overexpression, neuroinflammation and neuronal apoptosis. Moreover, 17β-estradiol ameliorated d-gal-induced AD-like pathophysiology, synaptic dysfunction and memory impairment in adult mouse brains. Interestingly, inhibition of SIRT1 with Ex527 (a potent and selective SIRT1 inhibitor) further enhanced d-gal-induced toxicity and abolished the beneficial effect of 17β-estradiol. Most importantly, for the first time, our molecular docking study reveals that 17β-estradiol allosterically increases the expression of SIRT1 and abolishes the inhibitory potential of d-ga. In summary, we can conclude that 17β-estradiol, in an ERα/SIRT1-dependent manner, abrogates d-gal-induced oxidative stress–mediated memory impairment, neuroinflammation, and neurodegeneration in adult mice.

## 1. Introduction

Aging involves the gradual loss of various physiological functions, impairment of the central nervous system, memory loss, cognitive disorders, and dementia [1]. Oxidative stress has been considered to be the main mediator in neurodegenerative disease and normal aging processes [2,3,4]. The well-known “free radical” theory of aging was presented by Denham Harman, based on free radicals and reactive oxygen species (ROS) produced as a result of normal metabolism as the primary cause of aging and aging-related degenerative diseases [5,6]. Several studies have reported that the accumulation of ROS, elevated oxidative stress, and neuroinflammation lead to impaired cellular function and are known to be associated with neuronal death in several age-related neurodegenerative disorders such as Alzheimer’s disease (AD) and Parkinson’s disease [7,8,9,10]. d-gal actose (d-gal) is a reducing sugar normally present in the body that is completely metabolized at normal concentrations by two enzymes, d-galactokinase and galactose 1-phosphate uridyltransferase. However, at high concentrations, it converts into aldose and hydrogen peroxide, resulting in the generation of superoxide anions and oxygen-derived free radicals that lead to ROS and oxidative stress [11,12]. It has been reported that chronic administration of d-gal for a period of 6–10 weeks results in ROS generation, inflammation, and malondialdehyde formation and lowers antioxidation and superoxide dismutase status in the brain, ultimately leading to age-related cognitive impairment [13,14,15].

Silent mating type information regulation 2 homolog 1 (SIRT1) is one of seven mammalian sirtuins that are class III histone deacetylase paralogues of yeast sir2 enzymes. It is a highly conserved class of nicotinamide adenine dinucleotide-dependent nuclear histone deacetylase and ADP-ribosyltransferase protein involved in the regulation of metabolism, stress responses, inflammation, circadian rhythm, and aging processes [16,17,18,19,20,21]. Transgenic mice overexpressing SIRT1 showed significant lifespan extension and phenotypes of delayed aging, whereas inhibiting SIRT1 expression in these mice abolished the effect of lifespan extension. Several studies reported that the estrogen receptor α (ERα)/SIRT1 complex functions as a suppressor of the *p53* gene in breast cancer [22,23]. SIRT1 deacetylates histones, signaling molecules, many transcription factors such as p53, forkhead box (FOXO), peroxisome proliferator-activated receptor gamma coactivator 1α (PGC1α), and liver X receptor (LXR), and inhibits the transactivational activity of nuclear factor kappa-light-chain-enhancer of activated B cells (NF-kB). Several studies have reported that SIRT1 reduces the level of oxidative stress and the extent of inflammation [24,25,26,27].

The steroid hormone 17β-estradiol is mainly synthesized by the enzyme aromatase in the ovary, as well as locally in tissues such as bone, adipose, and nerve tissues in both males and females. It has been reported that hippocampal neurons express E2-synthesizing enzyme P450 aromatase, which modulates synaptic function in vivo [28,29]. In recent decades, the brain has been considered as a “steroidogenic” organ because it expresses the molecules and enzymes that are required for the conversion of cholesterol into steroids such as progesterone, testosterone, and estradiol. The neurosteroid 17β-estradiol has an influence on various neurobiological process including cognition, stress, depression, body temperature, and sexual behavior [30,31]. Previously, it is reported that 17β-estradiol, via its antioxidant activity, free radical scavenging capability, and increased cell survival, inhibits neurotoxicity via estradiol receptors α and β (ERα and ERβ). In the brain, the roles of 17β-estradiol and its receptors are complex. Both ERα and ERβ bind to 17β-estradiol and activate estrogen-regulated target genes [32,33], and participates in the physiological regulation of inflammation. It has been documented that 17β-estradiol and estrogen receptor ligands exert anti-inflammatory effects in animal models [34].

The present study aims to investigate the protective effects of 17β-estradiol against d-gal-induced neurotoxicity. Our in vivo and in vitro results, along with a molecular docking approach, show that 17β-estradiol positively regulates SIRT1 via ERα, resulting in a reduction of oxidative stress, neuroinflammation, and neurodegeneration mediated memory loss in an adult mouse model.

## 2. Materials and Methods

### 2.1. Chemicals

The 17β-estradiol, d-galactose, 3-(4,5-Dimethylthiazol-2-yl)-2,5-Diphenyltetrazolium Bromide (MTT), Ex527 (potent and selective SIRT1 inhibitor), Tamoxifen and dimethyl sulfoxide (DMSO) were purchased from Sigma Aldrich (St. Louis, MO, USA) and Promega (Madison, WI, USA).

### 2.2. Animals and Drug Treatment

Wild-type C57BL/6N male mice (25–30 g body weight, eight weeks old) were purchased from Samtako Bio (Osan, South Korea). They were housed under a 12 h/12 h light/dark cycle at 23 °C with 60% ± 10% humidity and provided with water and food ad libitum. The mice were randomly placed into three groups: (1) control mice treated with saline as a vehicle for 10 weeks (C); (2) mice treated with d-galactose (100 mg/kg) for 10 weeks (d-gal); and (3) mice treated with d-gal (100 mg/kg) for six weeks followed by d-gal and estradiol (10 mg/kg) simultaneously for four weeks (d-gal + Est). The d-gal and estradiol were administered intraperitoneally. After treatment and behavioral analysis, the mice were sacrificed for further protein expression and immunofluorescences analysis. The animal experiments were performed in accordance with guidelines and regulations approved by the Institutional Animal Care and Use Committee (IACUC), Division of Applied Life Sciences, Gyeongsang National University, South Korea (Approval ID: 125).

### 2.3. Behavioral Analysis

After treatment, a behavioral study (n = 12–13 mice/group) was performed using a Morris water maze and Y-maze to evaluate spatial learning and memory, respectively. We performed the Morris water maze as described previously with some modification [35]. The apparatus is made of a circular water tank 100 cm in diameter and 40 cm in height. The tank was filled with water (23 ± 1 °C) to a depth of 15.5 cm and was made opaque by adding white ink. A transparent platform (10 cm in diameter, 20 cm in height) was hidden 1 cm below the water surface in one quadrant. The pool was located in a test room and contained various prominent visual cues. Each mouse received four training periods for four consecutive days. Latency to escape from the water (submerged escape platform) and swimming speed of each mouse was calculated. On day 5, a probe test was performed by removing the platform and allowing each mouse to swim freely for 60 s. At the probe trial, latency to the platform, swimming speed, time spent during swimming in the target quadrant and three other quadrants (right, left, and opposite), and number of platform crossings were measured. All data were recorded via a visual tracking system (SMART Panlab, Harvard Apparatus, Holliston, MA, USA).

### 2.4. Y-Maze Test

As previously described [36], the Y-maze was built from black-painted wood. Each arm of the maze was 50 cm long, 20 cm high, and 10 cm wide. Each mouse was kept at the center and was allowed to move freely through the maze for three 8 min sessions. The series of arm entries was visually observed and noted. Spontaneous alteration was defined as the mouse’s successive entry into the three arms in overlapping triplet sets. Alteration behavior (%) was calculated as successive triplet sets/total number of arm entries; 2 × 100.

### 2.5. Cell Culturing and Drug Treatment

Human neuroblastoma SH-SY5Y cells (obtained from the Korean Cell Line Bank) were cultured in 10% FBS (fetal bovine serum) and 1% penicillin/streptomycin-supplemented Dulbecco’s Modified Eagle Medium (DMEM) in a humidified 5% CO_2_ incubator at 37 °C [37]. The cells were treated with d-gal (10 mM i.e., 1.8 mg/mL), 17β-estradiol (1 µM i.e., 272.3 ng/mL), d-gal plus 17β-estradiol, d-gal plus Ex527 (80 uM i.e., 19.8 µg/mL), d-gal plus 17β-estradiol plus Ex527 and d-gal plus 17β-estradiol plus Tamoxifen (1 uM) for 4 h.

### 2.6. Cell Viability Assay

To analyze cell viability, an MTT assay was conducted according to the manufacturer’s instructions (Sigma). The cells were cultured in 96-well plates at a density of 1 × 10^4^ cells per well containing 100 µl DMEM. After attachment of the cells, the medium was replenished with fresh medium containing d-galactose (10 mM), 17β-estradiol (1 µM), d-galactose plus 17β-estradiol (10 mM + 1 µM), d-galactose plus Ex527 (10 mM + 80 uM), d-galactose plus 17β-estradiol plus Ex527 (10 mM + 1 µM + 80 uM) and Tamoxifen (1uM). The cells were further incubated for 3 h. The cells were then incubated for 4 h with MTT solution and the medium was replaced with DMSO. The absorbance was measured at 570 nm. The experiments were repeated in triplicate.

### 2.7. Oxidative Stress (ROS) Detection in Vitro

ROS assay was performed as previously described with slight modification [38,39]. The cells were cultured in 96-well plates in 200 μL DMEM containing 10% FBS and 1% penicillin/streptomycin in every well. Cells were incubated at 37 °C in a humidified incubator with 5% CO_2_ for 24 h. The next day, the medium was replaced by fresh medium and the cells were treated with d-galactose (10 mM), d-galactose plus 17β-estradiol (10 mM + 1 µM), d-galactose plus Ex527 (10 mM + 80 uM), and d-galactose plus Ex527 plus 17β-estradiol (10 mM + 80 uM + 1 µM) and Tamoxifen (1uM) for 4 h. Then 600 μM of 2′,7′-dichlorofluorescein diacetate (DCFDA) dissolved in DMSO/phosphate-buffered saline (PBS) was added to each well and incubated for 30 min. Plates were then read in an ApoTox-Glo^TM^ (Promega, Madison, WI, USA) at 488/530 nm.

### 2.8. Protein Extraction from Mouse Brain

For protein expression analysis, the mice (n = 7–8 mice/group) were sacrificed and brains were immediately removed and dissected carefully, frozen on dry ice, and stored at −80 °C. The tissues were homogenized in 0.2 M PBS with phosphatase inhibitor and protease inhibitor cocktail. The samples were then centrifuged at 10,000 g at 4 °C for 25 min. The supernatants were collected and stored at −80 °C.

### 2.9. Western Blot Analysis

Western blotting was performed as previously described [40]. Briefly, the protein concentrations in cell lysates and tissue homogenates were measured using a Bio-Rad protein assay (Bio-Rad Laboratories, Hercules, CA, USA). Equal amounts (20–30 μg) of protein were subjected to SDS-PAGE on 4%–12% Bolt^TM^ Mini Gels (Novex, Thermo Fisher Scientific, Waltham, MA, USA) and transferred to a Polyvinylidene fluoride (PVDF) membrane. Prestained protein ladders (GangNamstain^TM^, iNtRON Biotechnology, Burlington, NJ, USA) covering a broad range of molecular weights were used to detect the molecular weights of the proteins. The membranes were blocked in 5% skim milk/bovine serum albumin (BSA) to reduce nonspecific binding and incubated with primary antibodies (1:1000 dilution) at 4 °C overnight. After reaction with a horseradish peroxidase-conjugated secondary antibody as appropriate, the proteins were detected using an enhanced chemiluminescence detection reagent (Amersham Pharmacia Biotech, Uppsala, Sweden) according to the manufacturer’s instructions. The x-ray films were scanned, and the optical densities of the bands were analyzed through densitometry using the computer-based Sigma Gel program, version 1.0 (SPSS Inc., Chicago, IL, USA). Density values were calculated in arbitrary units (AU) relative to the untreated control.

### 2.10. Oxidative Stress (ROS) Detection in Vivo

ROS assay was performed as reported with slight modification, based on oxidation of 2,7-dichlorodihydrofluorescein diacetate (DCFH-DA) to 2,7-dichlorodihydrofluorescein [41,42]. The brain homogenates were diluted with ice-cold Locke’s buffer at 1:20 to get a final concentration of 5 mg tissue/mL. Then the reaction mixture (1 mL) including Locke’s buffer with pH 7.4, 0.2 mL of brain homogenate, and 10 mL of DCFH-DA (5 mM) was incubated for 15 min at room temperature to allow the DCFH-DA to be incorporated into any membrane-bound vesicles and the diacetate group cleaved by esterases. After 30 min of further incubation, the conversion of DCFH-DA to the fluorescent product DCF was measured using a spectrofluorimeter with excitation at 484 nm and emission at 530 nm. A parallel blank was used for background fluorescence (conversion of DCFH-DA in the absence of homogenate). ROS formation was quantified from a DCF standard curve and data are expressed as pmol of DCF formed/min/mg protein.

### 2.11. Determination of Lipid Peroxidation

Lipid peroxidation (LPO) quantification is essential to assess oxidative stress [43]. Free malondialdehyde (MDA), a marker of LPO, was measured in brain homogenate using lipid peroxidation (MDA) colorimetric/fluorometric assay kit (BioVision, USA, cat. #K739-100) according to the manufacturer’s protocol.

### 2.12. GSH Assays

The levels of total glutathione (GSH) and GSH/glutathione disulfide (GSSG) ratio were determined by using a fluorometric glutathione assay kit (BioVision Inc., Milpitas, CA, USA, cat. #K264–100), according to the manufacturer’s instructions.

### 2.13. Immunofluorescence

After behavioral studies, immunofluorescence staining was performed as previously reported [44,45]. Mouse brains were fixed by transcardial perfusion with ice-cold 4% paraformaldehyde. The brains were post-fixed for 72 h in 4% paraformaldehyde and then transferred to 20% sucrose for 72 h at 4 °C. The brains were mounted in optimal cutting temperature (OCT) compound (A.O., USA), frozen in liquid nitrogen, and sectioned (14 μm) in the coronal plane with a CM 3050C cryostat (Leica, Wetzlar, Germany). Tissue sections were prepared on Probe-On Plus charged slides (Thermo Fisher Scientific Inc., Waltham, MA, USA). In order to perform immunofluorescence analysis, slides were washed twice with 1× PBS followed by incubation with proteinase K solution at room temperature. After blocking in normal goat/rabbit serum (5% normal goat/rabbit serum, 0.3% Triton X-100, and PBS), primary antibodies (1:100 in PBS) were applied overnight at 4 °C. Fluorescence-based secondary antibodies in PBS (FITC and TRITC from Santa Cruz Biotech (Dallas, TX, USA), Cell Signaling Tech (Danvers, MA, USA), and Abcam (Cambridge, MA, USA) were applied at room temperature. Then 4′,6-diamidino-2-phenylindole (DAPI) was used to stain the nucleus. The slides were mounted with glass coverslips, and images were taken using a confocal microscope (FluoView FV 1000; Olympus, Tokyo, Japan).

### 2.14. Antibodies

The following primary antibodies were used in this study (Table 1). While goat anti-mouse and goat anti-rabbit horseradish peroxidase (HRP) secondary antibodies (dilution 1:10,000) were purchased from Santa Cruz Biotech (Dallas, TX, USA).

### 2.15. Molecular Docking Methodology

To predict the inhibition mode of d-gal against SIRT1 and the corresponding reactivation by 17β-estradiol, d-gal and 17β-estradiol were docked into the active and sirtuin-activating sites of SIRT1, respectively. The docking protocol implemented in the Molecular Operating Environment (MOE) program was used (Chemical Computing Group: Montreal, QC, Canada). As the crystal structure of the full-length SIRT1 is not available, we used the crystal structure of mini-hSIRT1, which contains only the functionally critical regions of the N and C terminal domains. The crystal structure of mini-hSIRT1 was obtained from the Protein Data Bank (PDB ID: 4zzi) and was prepared for docking by 3D protonation and energy minimization. A maximum of 10 conformations of each d-gal and 17β-estradiol in complex with SIRT1 was allowed to be saved using the default parameters of MOE (Placement: Triangle Matcher, Rescoring 1: London dG, Refinement: Forcefield, Rescoring 2: GBVI/WSA dG). The top-ranked conformations based on docking score were selected as the best predicted modes of inhibition and corresponding reactivation. The different conformations were ranked by scores from the generalized Born volume integral (GBVI)/weighted surface area (WSA) binding free energy calculation, which is the score of the last stage. The GBVI/WSA is a scoring function that estimates the free energy of the binding of ligand from a given pose. For all scoring functions, lower scores indicate more favorable poses. The resulting binding interactions between d-gal and SIRT1 and between 17β-estradiol and SIRT1 were observed using LigPlot implemented in MOE. The atoms of the receptor molecule away from the ligands were kept rigid during calculation, while atoms of the receptor in the locality of the ligand (the binding site) were kept flexible but were subjected to tether restraints to discourage gross movement.

### 2.16. Statistical Analysis

A computer-based Sigma gel system (SPSS Inc., Chicago, IL, USA) and the ImageJ program were used to analyze the integral optical density (IOD) and integrated density of scanned X-ray films of Western blot and immunofluorescence images, respectively. One-way ANOVA (analysis of variance) or 2-way ANOVA where appropriate, with Tukey’s post hoc test was used to determine the statistical significance (*p*-value) of the data. The density values of the data were expressed as mean ± standard error of the mean (SEM) of three independent experiments. *p*-values less than 0.05 were considered to be statistically significant; * *p* < 0.05, ** *p* < 0.01 indicates comparison between control and d-gal-treated groups; # *p* < 0.05, ## *p* < 0.01 indicates comparison between d-gal-treated and 17β-estradiol groups; and †, θ *p* < 0.05 indicates comparison between d-gal-treated and Ex527 groups.

## 3. Results

### 3.1. 17β-Estradiol Limits d-gal-Induced Oxidative Stress in Adult Mouse Brain

**Figure 1:**
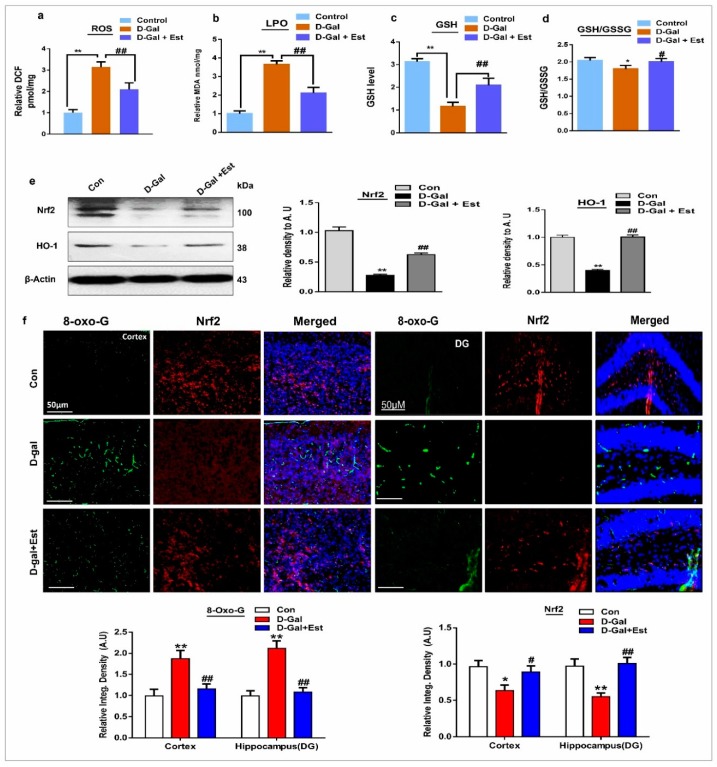
17β-estradiol attenuated the d-gal-induced increase in oxidative stress in adult mice brain. (**a**) The histogram of the reactive oxygen species (ROS) level in the brain homogenates of the adult mice. (**b**) A histogram represents the malondialdehyde (MDA) level in the brain homogenates of the adult mice. n = 7–8 mice/group, and the experiments were repeated in triplicate. (**c**) The representative histograms showing the glutathione (GSH) levels, and (**d**) GSH/glutathione disulfide (GSH/GSSG) ratio levels in the brain homogenates of the adult mice. (**e**) Shown are the Western blots results of nuclear factor erythroid 2-related factor 2 (Nrf2) and heme oxygenase-1 (HO-1) along with respective histograms in the brain homogenates of adult mice of all experimental groups. β-Actin was used as a loading control. (**f**) Representative images of immunofluorescence staining of colocalization of 8-Oxoguanine (8-OxoG) and Nrf2 in the cortex and hippocampus of the adult mice. n = 7–8 mice/group, and the number of experiments = 3. Magnification × 40. Scale bar = 100 μm. The data are shown here as a mean ± S.E.M. * significantly different from the control group; and # significantly different from d-gal-treated group, respectively; * & # *p* < 0.05, ** & ## *p* < 0.01.

First, we quantified d-gal-induced oxidative stress in the adult mouse brain by ROS and lipid peroxidation (LPO) assays. For this purpose, we performed respective ROS and LPO assays of mouse brain in all treated groups. The ROS and LPO assay results demonstrate that d-gal significantly increased oxidative stress in the mouse brain hippocampus. However, 17β-estradiol overcame this oxidative stress burden in the hippocampus (Figure 1a,b). In addition, we also examined glutathione (GSH and GSSG) levels. These assay results show that d-gal administration for 10 weeks significantly reduced cellular GSH content and the GSH/GSSG ratio in the adult mouse brain compared with saline-treated control (Figure 1c,d). Also, 17β-estradiol treatment significantly increased the cellular GSH content and GSH/GSSG ratio in d-gal-treated mice (Figure 1c,d).

Similarly, d-gal administration for 10 weeks significantly suppressed Nrf2 and HO-1 protein expression in the mouse brain hippocampus (Figure 1e). Western blot results reveal that 17β-estradiol injection significantly increased the expression of Nrf2 and HO-1 proteins in the hippocampus. Additionally, 17β-estradiol treatment increased the expression of antioxidant protein Nrf2 and reduced the 8-oxoguanine level in cortex and hippocampal dentate gyrus (DG) brain region tissues, as evident from the immunofluorescence co-localization results shown in Figure 1f.

### 3.2. 17β-Estradiol Reduced d-gal -Induced Neuroinflammation-Mediated Neurodegeneration via JNK/Akt/NF-κB/p53 Signaling in Vivo

**Figure 2:**
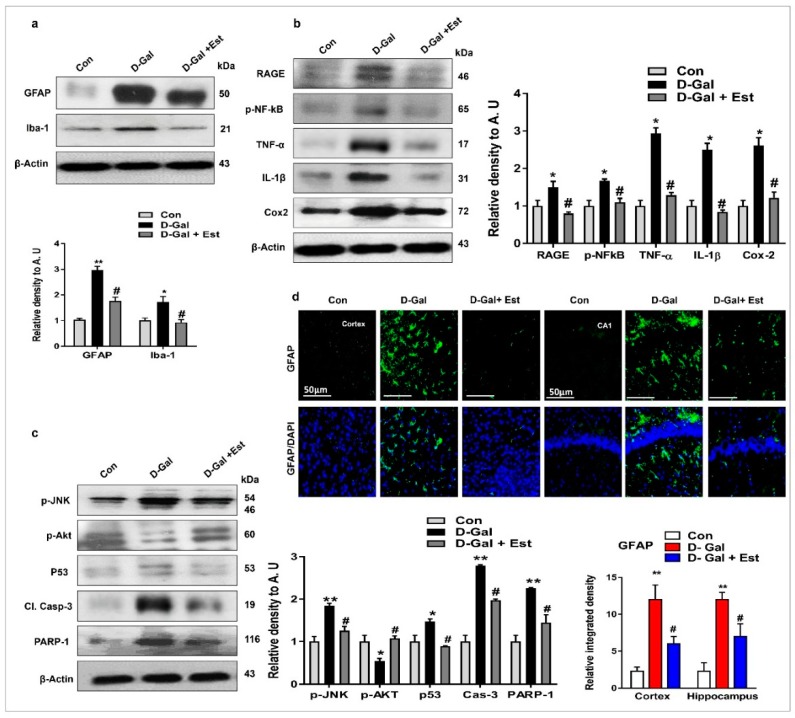
17β-estradiol attenuated the activated microglia, neuroinflammatory and neurodegenerative markers in d-gal -treated adult mice brain. (**a**) Western blot analysis of GFAP, Iba-1, (**b**) Receptor for advanced glycation end products (RAGE), phosphorylated nuclear factor kappa-light-chain-enhancer of activated B cells (p-NF-kB), Tumor necrosis factor alpha (TNF-α), Interleukin 1 beta (IL-1β), Cyclooxygenase (COX2) and (**c**) phosphorylated c-Jun N-terminal kinases 1 (p-JNK1), phosphorylated c-Jun N-terminal kinases 2 (p-JNK2), Protein kinase B (p-Akt), tumor suppressor protein (p53), cleaved caspases-3 (cl. Cas-3) and Poly(ADP ribose) polymerase-1 (PARP-1) in adult mice brain. The bands were quantified using ImageJ software, and the differences are depicted in the respective histogram. β-actin was used as a loading control. n = 7–8 mice/group, and the number of experiments = 3. (**d**) Representative images of immunofluorescence staining showing the GFAP in both the cortex and hippocampus of the adult mouse brain, respectively. n = 7–8 mice/group, and the number of experiments = 3. Magnification × 10. Scale bar = 50 μm. The data are expressed as the mean ± SEM. The data are presented relative to control values. Significance = *p* < 0.05. * Significantly different from the control saline-treated adult mice; # significantly different from the d-gal-treated adult mice, * & # *p* < 0.05, ** *p* < 0.01.

Several investigations have reported that d-gal-stimulated glial cells (both microglia and astrocytes) contribute to neuroinflammation-mediated neurodegeneration [46]. Similarly, c-Jun N-terminal kinase (JNK) has been described as an important stress kinase and is especially activated where ROS is induced [38], therefore, we investigated the phospho-JNK (p-JNK) protein and its downstream signaling molecules through Western blot. Western blot and immunofluorescence results revealed that d-gal treatment significantly activated microglia and astrocytes, resulting in increased expression of p-JNK in the adult mouse hippocampus (Figure 2a,c,d). Additionally, the expression of downstream signaling molecules of p-JNK, including p-Akt and associated inflammatory and neurodegenerative apoptotic markers such as RAGE, p-NF-ķB, TNF-α, IL-1β, COX2, p53, caspase-3, and PARP-1, in the adult mouse brain was also increased by d-gal administration compared to control mice (Figure 2b). Interestingly, 17β-estradiol co-treatment not only significantly reduced glial cell activation but also reversed the expression of p-JNK protein and its downstream signaling molecules as mentioned above (Figure 2a,c,d). The 17β-estradiol treatment activated the survival protein Akt, reduced the expression of apoptotic protein markers caspase-3, p53, and PARP-1, and significantly reduced neuronal cell death, as seen in the figure (Figure 2b–d).

### 3.3. 17β-Estradiol Reversed Alzheimer Disease-Like Pathology in d-gal-Treated Adult Mice

**Figure 3:**
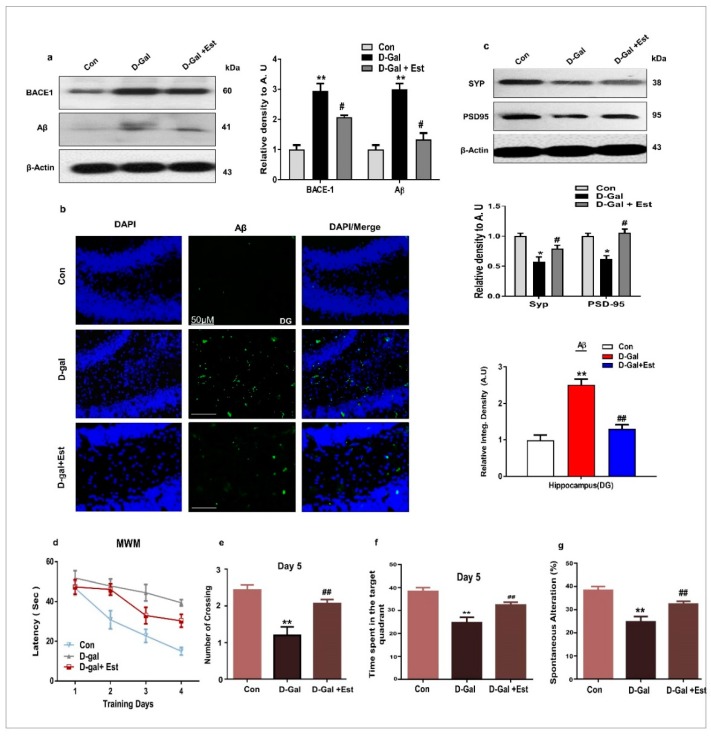
17β-estradiol reduced d-gal-induced amyloidogenic pathway upregulation and synaptic dysfunction along with memory impairment in adult mice brain. (**a**) Western blot analysis of Aβ proteins and beta-site amyloid precursor protein-cleaving enzyme-1 (BACE-1) in adult mice hippocampus after drug treatment. (**b**) Given are the representative images of immunofluorescence staining of Amyloid-β (Aβ) (n = 7–8 mice/group). The values represent averages from three independent experiments. For immunohistological quantitative analysis, the Image-J software was used. (**c**) Western blot analysis of the presynaptic Synaptophysin (SYP) and post-synapse (i.e., PSD95) along with their respective histograms. The density values were expressed in arbitrary units (AUs) as the mean ± S.E.M. (**d**) The mean escape latency time (s) histogram during the training days. (**e**) Number of platform crossings during the probe test. (**f)** Time spent in the target quadrant. (**g**) Spontaneous alternation behavior, given as a percentage (%) (n = 12–13 mice/group). The data are shown as a mean ± SEM. * significantly different from the control group and # significantly different from d-gal-treated group, respectively, * & # *p* < 0.05, ** & ## *p* < 0.01.

It was previously reported that oxidative stress plays an important role in both in AD brain and d-gal administered rodent models by increasing the activity of beta-site amyloid precursor protein (APP)-cleaving enzyme-1 (BACE-1) and Aβ protein [47]. For this reason, we analyzed the expression levels of BACE-1 and Aβ proteins after d-gal and 17β-estradiol treatment via Western blotting. The results indicate that 17β-estradiol significantly reduced the expression of BACE-1 and Aβ proteins in the brain homogenate of d-gal treated animals, as shown in Figure 3a. Additionally, 17β-estradiol significantly decreased the abundance of aggregated Aβ protein in the hippocampal DG region of adult mice in the experimental groups as evaluated by immunofluorescence (Figure 3b). Moreover, 17β-estradiol is also beneficial against d-gal-induced synaptic dysfunction by improving pre- and postsynaptic proteins synaptophysin (SYP) and post-synaptic density protein 95 (PSD95) (Figure 3c).

Finally, 17β-estradiol-treated mice showed better performance in the Morris water maze and Y-maze tests. In latency escape tests, all experimental groups showed a gradual decrease during the test days, but the d-gal-treated mice had longer latencies in comparison to the normal mice. On the other hand, 17β-estradiol-treated mice had shorter escape latencies. Additionally, 17β-estradiol-treated mice showed significantly more platform crossings and spent more time in the target quadrant compared to d-gal-treated mice (Figure 3d–f). Similarly, in the Y-maze task, the d-gal-treated mice had less spontaneous alterations than control mice, while 17β-estradiol-treated mice showed significantly increased spontaneous alterations, indicating improved short-term working memory (Figure 3g). All of these results support that 17β-estradiol could improve spatial learning and memory ability in d-gal-treated adult mice.

### 3.4. 17β-Estradiol Enhances the SIRT1 and Its Downstream Signaling Through ER-α against d-gal-Induced Neuroinflammation and Neurodegeneration

**Figure 4:**
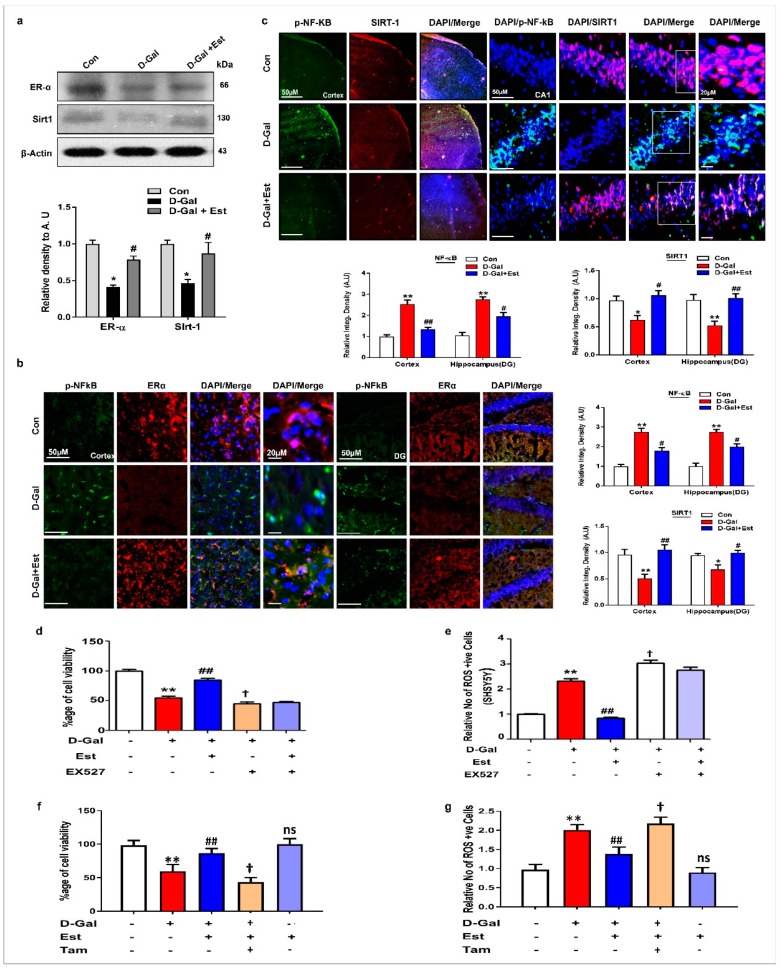
17β-estradiol stimulate estrogen receptor alpha (ER-α)/sirtuin 1 (SIRT1) signaling in d-gal treated mice and in vitro. (**a**) Shown are the western blot analysis of ER-α and SIRT1 in the adult mice brain homogenates of all three experimental groups. β-actin was used as a loading control. For protein band quantification ImageJ software was used. The density values were expressed in arbitrary units (AUs) as the mean ± SEM. (**b**,**c**) Given are the representative images of double immunofluorescence staining of ER- α, p-NF-kB and SIRT1 and in the hippocampal and cortical regions of the treated mice (n = 7–8 mice/group). The values represent averages from three independent experiments. For immunohistological quantitative analysis, the Image-J software was used. (**d**–**g**) Representative histograms of the relative absorbance of the MTT assay and ROS assay in human neuroblastoma SHSY5Y cells that were subjected to EX527, an inhibitor of SIRT1, Tamoxifen, an ERα inhibitor, and treated with d-gal and 17β-estradiol for 24 h. The number of experiments = 3. The data are expressed as the mean ± SEM. The data are presented relative to control values. *significantly different from the control group, # significantly different from d-gal -treated group and † significantly different from d-gal+Est group, respectively *, # & † *p* < 0.05, ** & ## *p* < 0.01.

According to previous knowledge that 17β-estradiol can bind to its own receptor, ERα [48,49,50,51], we determined whether 17β-estradiol activate SIRT1 through ERα dependent and independent manner. Our Western blot and immunofluorescence results showed that d-gal treatment reduce ERα and SIRT1 expression in adult mouse brain (Figure 4a–c). These finding reveal that 17β-estradiol activates SIRT1, either directly bound to ERα or indirectly (allosteric modulation).

To determine the beneficial effects of 17β-estradiol, we performed ROS and MTT assays in vitro using SHSY-5Y cells treated with d-gal and 17β-estradiol. The results show that d-gal significantly induced oxidative stress and cell death in SH-SY5Y cells after 24 h. However, co-treatment with 17β-estradiol not only reduced the oxidative stress, but also increased the cell viability of d-gal-treated cells (Figure 4d,e). To determine whether this effect of 17β-estradiol is ER α and SIRT1-dependent, we used Tamoxifen and EX527 as the inhibitor of ERα and SIRT1, respectively. Interestingly, on ERα and SIRT1 inhibition, the toxic effect of d-gal was more pronounced and further induced increased ROS and cell death, while it abolished the beneficial effects of 17β-estradiol treatment in SH-SY5Y cells, as shown in Figure 4d–g.

**Figure 5:**
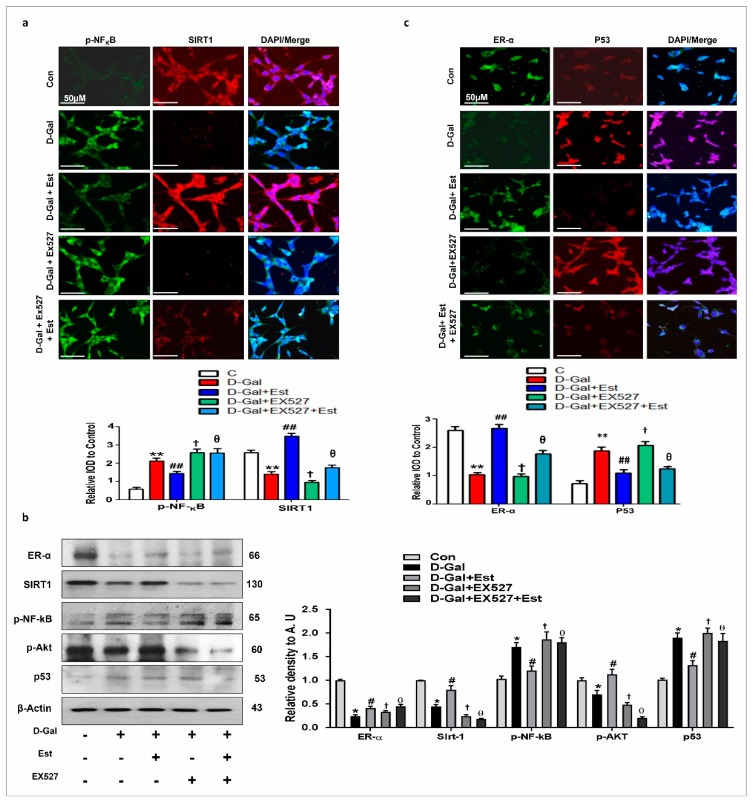
17β-estradiol in SIRT1 dependent manner abrogates d-gal-induced neuroinflammation and neurodegeneration in vitro. (**a**) The double immunofluorescence images of SIRT1 and p-NF-kB proteins by using SIRT1 inhibitor EX527 for 24 hr in SHSY5Y cells. (**b**) shown are the Western blot results along with their respective histograms in all experimental groups. The results were confirmed by using SIRT1 inhibitor EX527 for 24 hrs. β-actin was used as a loading control. For protein band quantification ImageJ software was used. The density values were expressed in arbitrary units (AUs) as the mean ± SEM. (**c**) The double immunofluorescence images of ER-α and p53 proteins by using SIRT1 inhibitor EX527 for 24 hr in SHSY5Y cells. Their differences are depicted in the respective histograms. The number of experiments = 3. The data are expressed as the mean ± SEM. * significantly different from the control group, # significantly different from d-gal-treated group, † significantly different from d-gal+Est group and θ significantly different from d-gal group (EX527) respectively *, #, † & θ *p* < 0.05, ** & ## *p* < 0.01.

Similarly, to determine whether the neuroinflammation and neurodegeneration induced by d-gal are also SIRT1-dependent, we blocked the activity of SIRT1 with EX527 in SH-SY5Y cells. Our results indicate that d-gal suppressed the expression of ERα, SIRT1, and p-Akt proteins, and significantly increased p-NF-kB and p53 proteins in SH-SY5Y cells (Figure 5a–c). Interestingly, 17β-estradiol in the absence of SIRT1 inhibitor reversed the changes induced by d-gal in SH-SY5Y cells, but in the presence of SIRT1 inhibitor, 17β-estradiol was unable to induce a beneficial effect (Figure 5a–c). This is also supported by double immunofluorescence staining between ERα and p53 and between SIRT1 and p-NF-kB in SH-SY5Y cells, showing that 17β-estradiol positively regulates ERα and SIRT1 and negatively regulates p53 and p-NF-kB, but when SIRT1 is blocked by its inhibitor, this greatly reduces its beneficial effects (Figure 5a,c).

### 3.5. 17β-Estradiol Reactivates SIRT1 after Deactivation by d-gal

**Figure 6:**
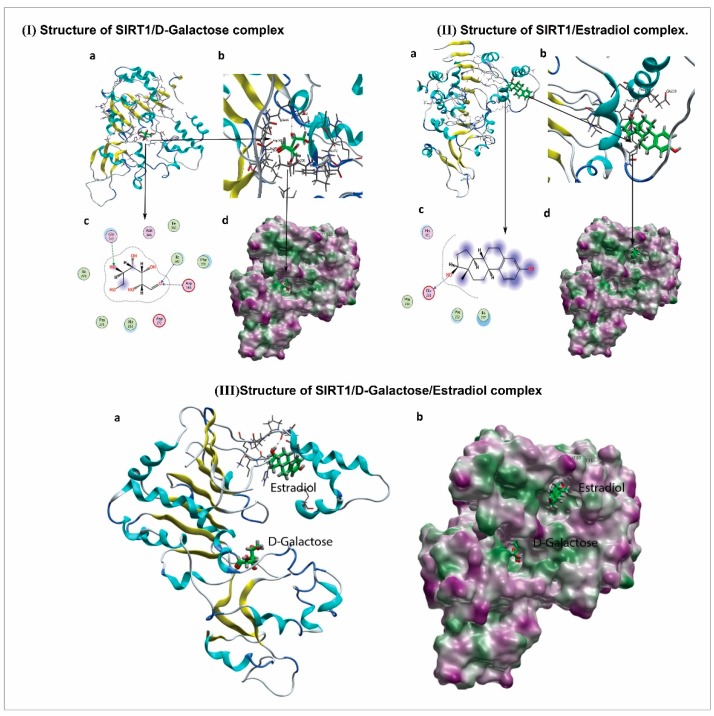
Structure and molecular docking of SIRT1/ d-gal /estradiol complex in ribbon diagram. (**I**) The structure of SIRT1/ d-galactose complex; (**II**) structure of SIRT1/estradiol complex; (**III**) structure of SIRT1/ d-galactose/estradiol complex.

Full-length SIRT1 contains three main structural regions: (1) the catalytic core, including residues 229–516, referred to as the central domain, creating the basic catalytic machinery; (2) the N-terminal region, containing residues 183–230, which facilitates sirtuin-activating compound binding and activation; and (3) the C-terminal regulatory segment peptide (641–665), which stabilizes the catalytic domain, resulting in more efficient deacetylase activity [52]. The SIRT1 catalytic domain contains a Rossmann-fold large lobe and a zinc-binding small lobe [47]. In the SIRT1– d-gal complex structure, the d-gal binds to the active site cleft between the large and small lobes (Figure 6I,a,d). The hydroxyl and carbonyl oxygen groups of d-gal form hydrogen bonds with the residues Gln345, Ile347, and Asp348. Thus, it was predicted from the docking conformation that the d-gal ligand was properly bound to the active site of SIRT1 by forming different intermolecular interactions.

The best docking conformation of d-gal showed three hydrogen bond acceptor interactions with a docking score calculated as −4.6994.Gln345 interacting with the OH group, with a bond length of 3.34 Å and bond energy of −1.0 Kcal/mol. Ile347 and Asp348 formed the other two hydrogen bond interactions with the carbonyl oxygen of the compound, with bond lengths of 3.05 Å and 3.16 Å and bond energies of −1.7 and −1.5 Kcal/mol, respectively (Figure 6I,b,c). In the docking study, significant binding interactions, binding energies, and docking scores were observed, which may reveal the good inhibitory potential of d-gal against SIRT1. To predict the interaction mode of 17β-estradiol for reactivating and increasing the enzymatic potency of SIRT1, 17β-estradiol molecule was docked over the N-terminal region containing residues 183–230 of the SIRT1 activating compound binding site in the presence of d-gal as an inhibitor.

The best docking conformation of 17β-estradiol on the SIRT1 activation binding site presented a good docking score of −4.6889 and extensive hydrophobic contact with the hydrophobic side chains of Pro231, Pro232, and Ile227, and only one hydrophilic interaction: hydrogen donor bond with Glu230 (Figure 6IIA–D). The bond length calculated for the predicted hydrogen bond was 3.18 Å and bond energy was −1.0 Kcal/mol. To examine the inhibitor d-gal binding status in the presence of 17β-estradiol activator, the tertiary complex (Figure 6IIIA,B) was subjected to energy minimization under the above-mentioned parameters. It was observed that the inhibition mode of d-gal was reduced to only one interaction: a hydrogen bond of comparatively low energy, −0.7 Kcal/mol.

## 4. Discussion

This study determined, for the first time, the therapeutic antioxidant efficacy of, and a detailed approach to, the mechanism of 17β-estradiol (estrogen) in minimizing d-gal-induced ROS-mediated neuroinflammation, neurodegeneration, and memory dysfunction in the adult male mouse brain. Moreover, during this study, we observed that 17β-estradiol activates SIRT1, which is involved in neuroprotection against d-gal-induced neurotoxicity both in vivo and in vitro. Additionally, we also showed through a docking study that d-gal deactivates SIRT1 and 17β-estradiol allosterically reactivates it. In this study, we used adult male mice to exclude the effects of varying levels of endogenous estrogen in female mice. Nevertheless, recent investigations provided evidence that menopause may not assure a steady estrogen level, which further results in enhanced aging and cognitive impairments in female mice [53,54].

d-gal is the main culprit in the production of oxidative stress and neurodegeneration in the central nervous system, which causes many complications [55,56]. In the present study, we demonstrated that d-gal induces oxidative stress, analyzed via ROS assay, lipid peroxidation assay (LPO), and glutathione assay, as well as the expression of 8-oxoguanine through immunofluorescence and Nrf2 and HO-1 levels via Western blotting. On the other hand, treatment of 17β-estradiol significantly abrogated the elevated levels of oxidative stress, as revealed by the decrease in the levels of ROS and LPO, and increase in the GSH/GSSG ratio in the 17β-estradiol-treated mice. Furthermore, the decrease immunoreactivity of 8-oxoguanine and the reversal in the expression levels of Nrf2 and HO-1 proteins in d-gal plus 17β-estradiol-treated mice confirmed that 17β-estradiol treatment is a potent anti-oxidant agent against d-gal -induced oxidative stress in the brain.

Oxidative stress is strongly implicated in neuroinflammation and the activation of microglia and astrocytes [35]. Previous studies reported that d-gal treatment activates microglia and astrocytes and other inflammatory mediators in animal brains [46]. It is assumed that d-gal treatment would activate neuroinflammation via elevated oxidative stress in the d-gal-treated mouse brains. Furthermore, it is confirmed that oxidative stress and neuroinflammation are critical players in neuronal apoptosis and neurodegeneration. d-gal treatment induces neuronal apoptosis and neurodegeneration, which may involve d-gal-induced elevated oxidative stress and neuroinflammation in the mice brain. Another study demonstrated that oxidative stress–mediated RAGE and NF-kB activation resulted in amyloid precursor protein (APP) fragmentation and Aβ production [57]. In this regard, some authors have claimed that d-gal is responsible for the upregulation of RAGE along with glial cells and NF-kB activation, inducing neuroinflammation and neurodegeneration in the brain [58,59]. Both histological and immunofluorescence analysis in our mouse model of d-gal treatment revealed that d-gal treatment significantly evoked neuroinflammation, as indicated by increase in gliosis and the expression of other pro-inflammatory mediators such as p-NF-_K_B, TNF-α, IL-1β and Cox-2 in the d-gal treated mice. It was interesting to show that 17β-estradiol treatment significantly reversed the effects and inhibited neuroinflammation in the d-gal treated mice. Other studies have shown the neuroprotective effects of 17β-estradiol treatment in terms of a significant decrease in reactive oxygen species (ROS) and nitric oxide (NO) production, and a notable decrease in gliosis levels in different mouse models [49,60,61]. Furthermore, our data confirmed that d-gal treatment induces neuronal apoptosis as revealed by increase expression of active JNK and other apoptotic markers such as Bax, cleaved caspase-3, p53, PARP-1 and the decreased expression of pro-survival Bcl-2. However, 17β-estradiol treatment significantly ameliorated neuronal apoptosis in the mice brain. These observations suggest that the treatment of 17β-estradiol might reduce neuronal cell apoptosis and neurodegeneration via reduction of oxidative stress and inhibition of neuroinflammation in the mouse brain.

Oxidative stress has been reported to increase BACE-1 expression, ultimately resulting in increased Aβ generation in humans as well as in AD mouse models [62,63,64]. Some reports favor our current findings that animals administered d-gal displayed increased expression of both BACE-1 and Aβ [45,58]. We therefore examined the AD-like pathological markers in the d-gal treated mice brains. Our results clearly indicated that d-gal treatment significantly increased the amyloidogenic pathway and increased the expression of beta-site amyloid precursor protein cleaving enzyme (BACE-1). It is this abundant Aβ produced in d-gal-treated mice due to oxidative stress that induces neurodegeneration and cognitive impairment [65]. On the other hand, we found that 17β-estradiol treatment significantly reduced amyloidogenic pathway and reduced the level of BACE-1. Previous studies demonstrated that 17β-estradiol depletion results in increased microglial activation and β-amyloid production. Moreover, long-term 17β-estradiol treatment inhibited NF-ĸB and microglial activation, further protecting memory impairment through the regulation of amyloid beta [66,67]. Our results reveal that d-gal administration or treatment in vitro can cause neurotoxicity leading to neurodegeneration in animals. Additionally, 17β-estradiol treatment either in vivo or in vitro minimized d-gal-induced neurodegeneration. Most importantly, we showed that d-gal is responsible for oxidative stress and neuroinflammation-mediated neurodegeneration in the adult mouse brain. All of these events suggest that activated BACE1 causes the fragmentation of APP into toxic Aβ production and accumulation in the brains of adult mice, leading to synaptic and memory dysfunction, making this model a perfect match with AD pathology. We used 17β-estradiol as a neuroprotective agent, and interestingly, it activated SIRT1 and its downstream signaling molecules to reduce d-gal-induced oxidative stress, neuroinflammation, and neurodegeneration-mediated synaptic and memory dysfunction in adult mouse brains.

Previous studies demonstrated that 17β-estradiol can bind to its own receptor, ERα [48,49,50,51]. We determined whether 17β-estradiol can bind to its receptor and then activate SIRT1. Our immunoblot and immunohistological results obtained from both in vivo and in vitro studies clearly demonstrated that d-gal treatment reduced ERα and SIRT1 expression in adult mice brains as well as in neuronal cells suggesting that d-gal-induced oxidative stress might be involved in the suppression of ERα, SIRT1 and the deregulated downstream signaling molecules. We further examined the expression levels of the downstream signaling molecules via immunoblot and immunohistological analysis. The results clearly demonstrated that d-gal treatment significantly activates NF-_K_B and apoptotic markers in the mice brain as well as in cell lines. Interesting treatment of 17β-estradiol significantly reversed the depressed expression levels of ERα, SIRT1 and inhibited activated NF-_K_B as well as apoptotic neurodegeneration both in vivo and in vitro.

Several studies have reported that sirtuin-activating compounds (STACs) modulate SIRT1 activity to deacetylate its substrate protein(s). In this regard, the results of other studies are related to our current findings, as Hubbard et al. reported that STACs potentiate the allosteric activation of SIRT1 by physical interaction with Glu230 in its N-terminal domain [68]. In parallel, our molecular docking analysis also suggests that 17β-estradiol binds to the N-terminal domain of SIRT1 and forms a hydrogen bond with Glu230, even in the presence of d-gal. Therefore, we believe that 17β-estradiol induces allosteric activation of SIRT1 and abolishes the inhibitory potential of d-gal. Our docking study is in agreement with our wet lab results and reveals for the first time that d-gal is involved in the inhibition of SIRT1, while 17β-estradiol allosterically modulates and is potentially responsible for the activation of SIRT1.

Previous reports also demonstrated that SIRT1 reduces the level of oxidative stress and the extent of inflammation [24,25,26,69]. SIRT1 is known as a longevity-associated protein and serves as a potential pharmacological target to increase the lifespan of humans and stands at the front line against cognitive decline, neurodegenerative disease, and aging. SIRT1 is crucial for synaptic plasticity in neurons and memory function, and also induces neuronal protection in neurodegenerative diseases and memory impairment [70]. Likewise, Sun et al. [71] proved through the use of siRNA of SIRT1 that it plays a crucial role in AD pathology. Additionally, Albani et al. [72] confirmed that resveratrol, an SIRT1 activator, also possessed neuroprotective effects in an AD model. Similarly, Han et al. reported that SIRT1 activity regulated through estradiol delayed the cell aging process [73]. Moreover, some studies correlate with our study, showing that SIRT1 is involved in exerting neuroprotection against d-gal-induced oxidative stress and apoptosis both in vivo and in vitro [74,75]. Taken together, these studies show that SIRT1 may be a critical regulator of aging, exerting a pivotal role in memory and behavior, and is closely related to cognitive decline in aging and neurodegenerative diseases.

## 5. Conclusions

Our current findings demonstrated that 17β-estradiol abrogated d-gal-induced oxidative stress, neuroinflammation, and neurodegeneration that caused synaptic and memory dysfunction in the brains of d-gal -treated adult male mice. Our suggested approach of 17β-estradiol’s neuroprotective effects against d-gal is dependent on SIRT1 and their downstream signaling molecules. Furthermore, we hope to extend this work in ovariectomized female mice and male mice for comparative analysis to evaluate the beneficial effects of estradiol against age-related deficits in cognitive performance.

## Figures and Tables

**Table 1:** List of primary antibodies and their information used in this study.

Antibody	Catalog	Application (Conc.)	Host	Manufacturer
anti-β-actin	sc-47,778	WB (1:1000)	Mouse	Santa Cruz Biotech (Dallas, TX, USA)
anti-Nrf2	sc-722	WB/IF (1:1000/1:100)	Mouse	Santa Cruz Biotech (Dallas, TX, USA)
anti-Akt	sc-514032	WB (1:1000)	Mouse	Santa Cruz Biotech (Dallas, TX, USA)
anti-HO1	sc-136961	WB (1:1000)	Mouse	Santa Cruz Biotech (Dallas, TX, USA)
anti-Iba-1	sc-32725	WB (1:1000)	Mouse	Santa Cruz Biotech (Dallas, TX, USA)
anti-GFAP	sc-33673	WB/IF (1:1000/1:100)	Mouse	Santa Cruz Biotech (Dallas, TX, USA)
anti-IL-1β	sc-32294	WB (1:1000)	Mouse	Santa Cruz Biotech (Dallas, TX, USA)
anti-TNF-α	sc-52746	WB (1:1000)	Mouse	Santa Cruz Biotech (Dallas, TX, USA)
anti-p-NF-κB	sc-136548	WB/IF (1:1000/1:100)	Mouse	Santa Cruz Biotech (Dallas, TX, USA)
anti-p-JNK	sc-6254	WB (1:1000)	Mouse	Santa Cruz Biotech (Dallas, TX, USA)
anti-PSD-95	sc-71,933	WB (1:1000)	Mouse	Santa Cruz Biotech (Dallas, TX, USA)
anti-PARP-1	sc-8007	WB (1:1000)	Mouse	Santa Cruz Biotech (Dallas, TX, USA)
anti-Cl-Casp-3	sc-7272	WB (1:1000)	Mouse	Santa Cruz Biotech (Dallas, TX, USA)
anti-Cox2	sc- 7951	WB (1:1000)	Rabbit	Santa Cruz Biotech (Dallas, TX, USA)
anti-RAGE	sc-365154	WB (1:1000)	Mouse	Santa Cruz Biotech (Dallas, TX, USA)
anti-P53	sc-126	WB/IF (1:1000/1:100)	Mouse	Santa Cruz Biotech (Dallas, TX, USA)
anti-BACE1	sc-33711	WB (1:1000)	Mouse	Santa Cruz Biotech (Dallas, TX, USA)
anti-Aβ	sc-28365	WB/IF (1:1000/1:100)	Mouse	Santa Cruz Biotech (Dallas, TX, USA)
anti-ERα	ab75635	WB/IF (1:1000/1:100)	Rabbit	Abcam (Cambridge, MA, USA)
anti-SIRT1	#9475	WB/IF (1:1000/1:100)	Rabbit	Cell Signaling Tech (Danvers, MA, USA)
8-OXO-G	MAB3560	IF (1:100)	Mouse	Millipore, USA (Billerica, MA, USA)

(WB: Western blotting, and IF: immunofluorescence).
